# Imaging of hepatocellular carcinoma recurrence after liver transplantation

**DOI:** 10.1186/s13244-023-01425-6

**Published:** 2023-05-15

**Authors:** Giuseppe Mamone, Settimo Caruso, Mariapina Milazzo, Giorgia Porrello, Ambra Di Piazza, Giovanni Gentile, Vincenzo Carollo, Francesca Crinò, Gianluca Marrone, Gianvincenzo Sparacia, Luigi Maruzzelli, Roberto Miraglia, Salvatore Gruttadauria

**Affiliations:** 1 Radiology Unit, Department of Diagnostic and Therapeutic Services, IRCCS ISMETT (Mediterranean Institute for Transplantation and Advanced Specialized Therapies), Via Tricomi 5, 90127 Palermo, Italy; 2 Department for the Treatment and Study of Abdominal Diseases and Abdominal Transplantation, IRCCS ISMETT (Mediterranean Institute for Transplantation and Advanced Specialized Therapies), Palermo, Italy

**Keywords:** Recurrence, Hepatocellular carcinoma, Liver transplantation

## Abstract

**Abstract:**

Liver transplantation (LT) provides the highest survival benefit to patients with unresectable hepatocellular carcinoma (HCC). The Milan criteria have been developed for the selection of LT candidates with the goal of improving survival and maintaining an acceptable risk of HCC recurrence. Despite this, recurrence of HCC after LT occurs in up to 20% of cases and represents a major concern due to the poor prognosis of these patients. Furthermore, several extended criteria for the selection of LT candidates have been proposed to account for the growing demand for organs and the resultant increase in the risk of HCC recurrence. Radiologists should be aware that HCC can recur after LT with multiple organ involvement. Knowledge of the location and radiologic appearance of recurrent HCC is necessary to ensure the choice of the most appropriate therapy. This paper aims to comprehensively summarize the spectrum of HCC recurrence after LT and to examine and discuss the imaging features of these lesions.

**Graphical Abstract:**

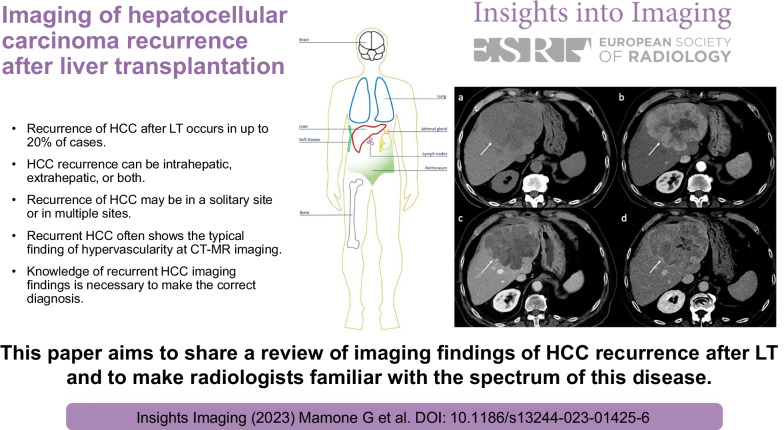

**Critical relevance statement:**

This paper aims to share a review of imaging findings of HCC recurrence after LT and to make radiologists familiar with the spectrum of this disease.

## Key points

- Recurrence of HCC after LT occurs in up to 20% of cases.
- HCC recurrence can be intrahepatic, extrahepatic, or both.
- Recurrent HCC may be in a solitary site or in multiple sites.
- Recurrent HCC often shows the typical finding of hypervascularity at CT-MR imaging.
- Knowledge of recurrent HCC imaging findings is necessary to make the correct diagnosis.

## Background

Hepatocellular carcinoma (HCC) is the primary liver tumor, classified as the sixth most frequent cancer and the third cause of cancer-related death [1].

Countries in the Western Hemisphere show that most HCCs evolve together with liver cirrhosis due to long-term viral hepatitis, alcohol misuse, and latterly, subsequent to complications of non-alcoholic steatohepatitis (NASH) [2, 3].

A liver transplant (LT) is the best care option since it treats both the cancer and the primary liver illness (the main risk factor for the evolution of new cancers) [4, 5]. Unfortunately, there are not sufficient available grafts to cover the demand in patients with cirrhosis needing a new liver. As a result, physicians are forced to allocate organs efficiently to patients with a greater survival benefit. In accordance with the Milan criteria, published in 1996 by Mazzaferro et al. [6], LT should be performed in patients with unresectable HCC characterized by a single tumor ≤ 5 cm or three tumors all ≤ 3 cm. Furthermore, macrovascular invasion and extrahepatic spread must be absent. When these criteria are applied, LT allows a 4-year survival rate of 75–85% and recurrence-free survival of 83% [6]. Nevertheless, HCC recurs in 8% of cases, becoming the first cause of death [6]. In other later studies on LT patients following the Milan criteria, recurrent HCC is found in 10–20% of cases [7–9]. Further studies show that HCC recurrence happens despite the application of rigid selection criteria for LT and comes from the growth of hidden metastases over time (months or years) after transplant (most likely) or from the release of circulating HCC cells during the procedure (less likely) [10, 11]. Recently, a discussion arose on the possibility to extend the Milan criteria for LT. While this may result in an initial decrease in patient mortality caused by cancer progression, it could be accompanied by an increased rate of cancer recurrence after LT [12]. Risk factors for recurrence are connected to cancer, the recipient and the donor, and immunosuppressive therapy required after transplant. Assuming that LT patients can develop HCC recurrence, radiologists should be aware of this occurrence during follow-up. This paper aims to share a review of imaging findings of HCC recurrence after LT and to make radiologists familiar with the spectrum of this disease.

## Risk factors associated with hcc recurrence

The development of recurrent HCC after LT seems to depend on several factors or causes. Risk factors involved in HCC recurrence may be split into those related to the tumor and those unrelated to the tumor (Table 1) [3]. It should be considered, however, that some of the factors mentioned have not been validated in prospective studies nor in large numbers of patients.

**Table 1:** Risk factors associated with HCC recurrence

Risk factors related to the tumor
*Morphology*
Tumor burden: number and size of the nodules (Milan criteria, extended criteria)
Presence of macrovascular invasion
Presence of extrahepatic spread
*Histology*
Poor tumor differentiation
Microvascular invasion
Genetic signature (CK19, etc.)
*Serum markers*
Increased AFP serum level (Metrotickets 2.0 model, etc.)
Increased DCP serum level
Inflammatory markers (increased NLR; increased PLR)
*Response to anticancer treatments (TACE, RE, TA, surgery)*

Risk factors unrelated to the tumor
*Characteristics of the recipient*
Older age
Obesity
Severity of underlying disease
Immunological status
*Liver graft*
Older age of the donor
LDLT versus DDLT
Steatosis
Prolonged cold ischemia and ischemia–reperfusion injury
*Immunosuppression after LT*
CNI (tacrolimus, cyclosporine)
mTOR inhibitors (sirolimus and everolimus)

*AFP* α-fetoprotein, *DCP* des‐*γ*-carboxy prothrombin, *NLR* neutrophil-to-lymphocyte ratio, *PLR* platelet-to-lymphocyte ratio, *TACE* transarterial chemoembolization, *RE* radioembolization, *TA* thermoablation, *LDLT* living donor liver transplantation, *DDLT* deceased donor liver transplantation, *LT* liver transplantation, *CNI* calcineurin inhibitors, *mTOR* inhibitors of the mammalian target of rapamycin

Risk factors related to the tumor are as follows:

- Morphology;
- Histology;
- Serum markers;
- Response to anticancer treatments.

Risk factors unrelated to the tumor are as follows:

- Characteristics of the recipient (age, obesity, severity of underlying liver disease, immunological status);
- Liver graft (old age of the donor, percentage of steatosis, prolonged cold ischemia and ischemia–reperfusion injury, living versus cadaveric donor);
- Immunosuppression after LT (CNI, mTOR inhibitors).

Recent risk prediction models of HCC recurrence after LT have been developed after extensive studies of the above-mentioned risk factors. Risk prediction models are classified as follows:

- Preoperative models take into consideration the morphological, serological, and histological characteristics of HCC. These models may be utilized to identify LT candidates by evaluating the future risk of developing recurrent HCC.
- Postoperative models consider histological risk factors for HCC based on the explanted liver [i.e., cancer grading and eventual presence of microvascular invasion (MVI)].
- General risk models are made from a combination of both pre- and postoperative risk factors. Consequently, they cannot be adopted to select HCC patients for transplantation. On the other hand, they can suggest the optimal post-LT surveillance or be used to design clinical trials on neo-adjuvant therapies [13].

## Imaging of HCC recurrence

**Fig. 1:**
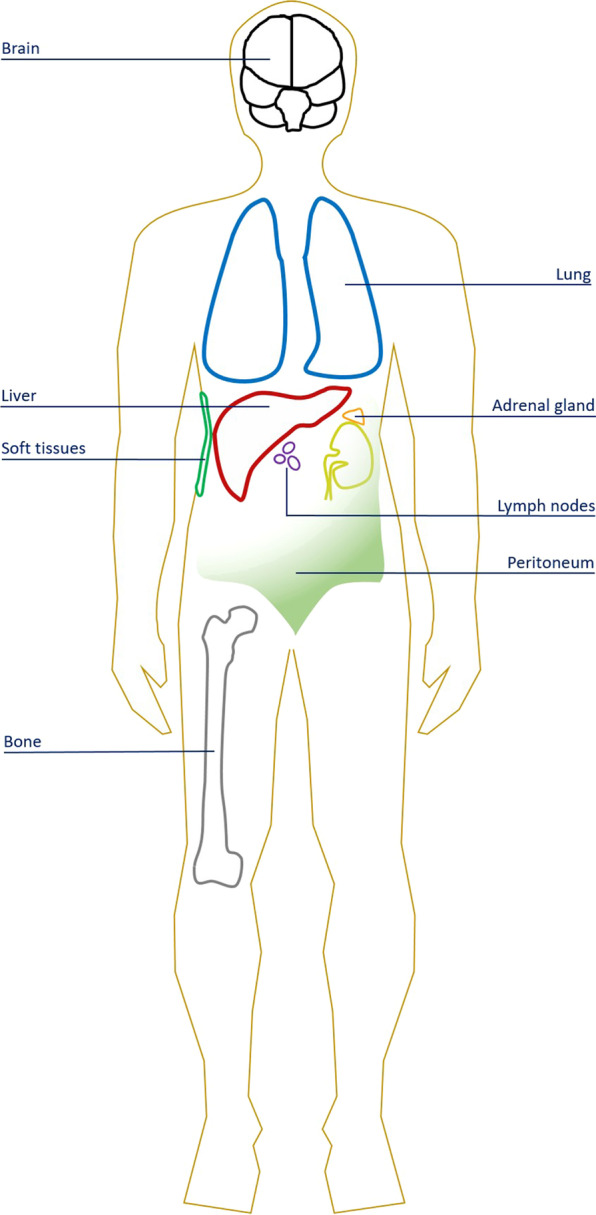
Sites of hepatocellular carcinoma recurrence after liver transplantation. Tumor recurrence is mainly extrahepatic, particularly in the lungs (40–60%) and bones (25–30%). Other extrahepatic locations of recurrent HCC are the adrenal glands (~ 10%), lymph nodes (~ 10%), peritoneum (~ 10%), brain (~ 4%), and rarely soft tissues/muscles of the thoracic and abdominal wall. Extrahepatic-only recurrence accounts for 50–60% of cases. Liver and extrahepatic recurrence occurs in 30–40% of cases. Liver-only recurrence accounts for 15–40% of cases

Recurrence of HCC following LT is very tricky, not only due to the potential loss of limited graft resources but also because of the added risk of faster tumor progression in immunocompromised hosts. Currently, there are no incontrovertible guidelines recommending the surveillance of HCC recurrence after liver transplantation. The main issues are whether surveillance for HCC recurrence provides a worthy contribution to care and whether it is cost-effective. The evidence suggests that surveillance would be valuable for patients. A prospective search for tumor recurrence should probably include body CT scans every 6 months, for at least the first 3 years after LT. Thereafter, CT could be done yearly [10]. Indeed, peak cancer recurrence occurs around 2–3 years post-transplant [10]. Approximately 75% of tumor recurrence occurs during the first 2 years after LT, and only 10% is identified after the fourth year [14]. The average time to recurrence varies from 1 to 2 years following LT, and the median survival from the time of diagnosis is about 1 year. Most authors only evaluate cancer developed within the first year after LT as early recurrence. Patients with HCC recurrence within the first year have poor outcomes [10]. Patients with late HCC recurrence, with low serum AFP levels, and who have the possibility to be aggressively treated (surgical resection) achieve a 50% 5-year survival rate after HCC recurrence has been identified [10]. HCC spreading can occur in many ways before or during LT: hematogenous spread, direct extension, lymphatic spread, or rupture of a lesion with intraperitoneal implantation of tumor cells into peritoneal or omental surfaces. Furthermore, HCC tends to invade veins directly, mostly the portal, followed by the hepatic, and, lastly, the inferior vena cava. HCC recurrence can be intrahepatic, extrahepatic, or both [14–22] (Fig. 1). Tumor recurrence is frequently extrahepatic, particularly in the lungs (40–60%) and bones (25–30%) [10]. Other extrahepatic locations of recurrent HCC are the adrenal glands (~ 10%), lymph nodes (~ 10%), peritoneum (~ 10%), brain (~ 4%) [14], and rarely soft tissues/muscles of the thoracic and abdominal wall. Extrahepatic-only recurrence causes 50–60% of cases. Both liver and extrahepatic recurrences occur in 30–40% of cases. Liver-only recurrence represents 15–40% of cases. Recurrent HCC may be in a solitary site or in multiple sites. Depending on the site, recurrent HCC often shows the typical finding of hypervascularity with enhancement on arterial phase contrast-enhanced CT and MR imaging. Detection of new intra- or extrahepatic lesions in patients after LT often brings about a differential diagnosis that, in some patients, may be narrowed down only after biopsy. In the remaining portion of patients, macroscopic development of new lesions together with radiologic appearance, a rising serum AFP level or negative findings for a secondary malignancy, may be acceptable as proof of recurrent HCC.

### Liver recurrence

**Fig. 2:**
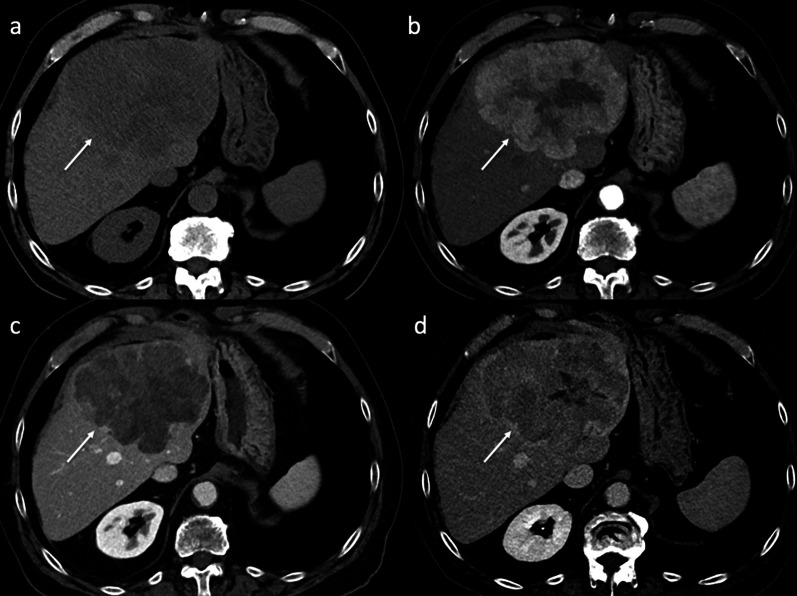
Liver recurrence. Unenhanced (**a**) and dynamic contrast-enhanced (**b**–**d**) axial CT imaging shows recurrent HCC (arrows) in both the hepatic lobes. The lesion shows the typical pattern of HCC: hypervascularity on the arterial phase (**b**), with washout in the portal venous (**c**) and 3-min late phases (**d**)

The liver is one of the most common sites of recurrent HCC after LT. Liver recurrence may occur alone (15–40% of cases) or be associated with extrahepatic lesions (30–40% of cases). Usually, a recurrent HCC has similar attributes to its primary tumor. Radiologically, it shows the typical pattern of hyperenhancement on arterial phase contrast-enhanced CT and MR imaging followed by washout on portal and/or equilibrium phases (Fig. 2). However, despite the detection of HCC in native livers, there is a lack of literature on magnetic resonance imaging (MRI) findings in patients with tumor recurrence after LT. In order to standardize the acquisition, interpretation, reporting, and data collection of liver imaging, the American College of Radiology developed the Liver Imaging Reporting and Data System (LI-RADS) [19]. LI-RADS is used in clinical practice for patients at high risk of HCC and includes post-transplant patients with HCC recurrence. This system categorizes hepatic lesions from LR-1 (definitely benign) to LR-5 (definitely HCC) based on the imaging findings aiding the diagnosis of HCC. The diagnosis of small recurrent tumors in the liver may be difficult because of the atypical imaging findings that they may show. Hepatobiliary MRI is better than CT for detection and differentiation of small recurrent HCC. A recent study by Kim et al. [20] shows the imaging findings of recurrent HCC in the hepatic graft after LT based on hepatocyte-specific contrast agent MR imaging. The authors show an atypical enhancement pattern of HCCs using gadoxetic acid-enhanced MR imaging, characterized by the absence of washout on the portal or delayed phase in most lesions. Furthermore, recurrent HCCs in transplanted livers show more cholangiocarcinoma-like features than tumors arising from the native liver. In these cases, the lesions show a peripheral rim enhancement on the arterial phase and a target appearance on hepatobiliary MR images. Of note is that the authors do not find a remarkable outcome difference related to the enhancement pattern in either subgroup. These atypical patterns could be explained by two hypotheses: First, most HCC recurrences after LT may be caused by hematogenous metastasis rather than the known sequential multistep process of hepatocarcinogenesis in patients with cirrhosis [20]; second, the immunosuppression therapy for organ transplant recipients and the changes in liver parenchymal structure and vascularity can cause some degree of hemodynamic variability and modify the tumor enhancement pattern [20, 21]. Liver lesions after LT could indicate recurrent HCC, but, based on radiologic findings, it is needed to clearly distinguish between benign hemangiomas transferred in the liver allograft, metastases from a different primary malignancy, intrahepatic hematomas or complex bilomas after percutaneous biopsy or cholangiography, infarcts, abscesses, and occasional solid-organ post-transplantation lymphoproliferative disorder (PTLD). The differential diagnosis also includes hepatic pseudolesions, which may be caused by changes in intrahepatic hemodynamics with the appearance of abnormal imaging findings on arterial phase contrast-enhanced imaging. For example, a shunt between the hepatic artery and portal vein and a transient hepatic attenuation/intensity difference can seem like a hypervascular tumor.

### Lymph node recurrence

**Fig. 3:**
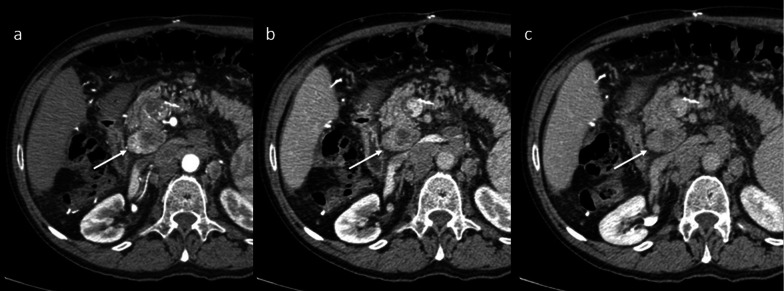
Lymph node recurrence. Axial contrast-enhanced CT images on the arterial phase (**a**), portal venous phase (**b**), and 3-min late phase (**c**) exhibit a metastatic portacaval lymph node with a central necrotic area, showing the typical pattern of HCC: inhomogeneous enhancement on the arterial phase and washout in the 3-min late phase

**Fig. 4:**
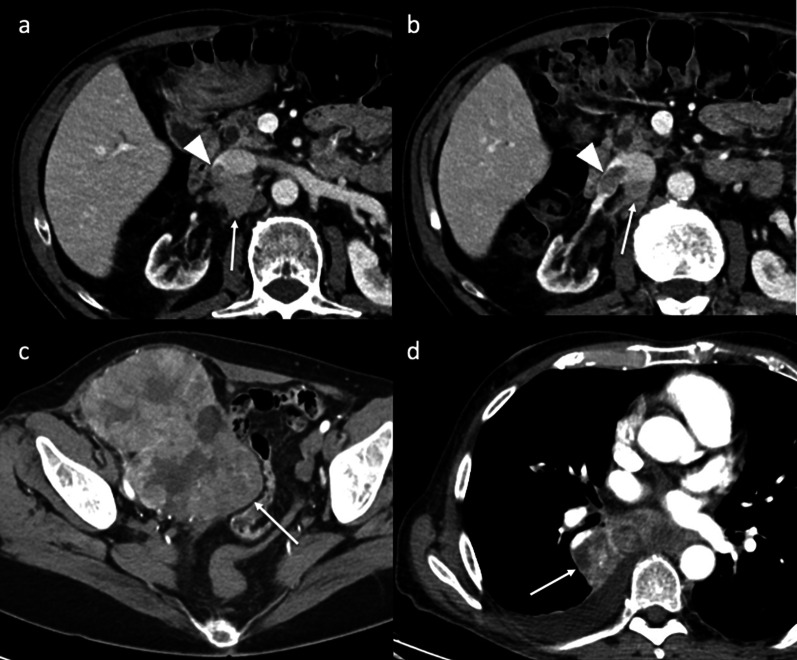
Lymph node recurrence. Axial contrast-enhanced CT images on the portal venous phase (**a**, **b**) show paracaval metastatic lymph node (arrows) infiltrating the inferior vena cava and the right renal vein (arrowheads). In two different patients, arterial phase CT images exhibit a huge hypervascular metastatic lymph node in the pelvis (**c**) and confluent metastatic lymph nodes with hyperenhancement in the posterior mediastinum (arrows). Notice the presence of hyperenhancement on the arterial phase, typical of recurrent HCC

Lymphatic spread of HCC was discovered in patients who underwent LT. The most frequent sites are regional, particularly in the hepatic hilum and the portacaval, paracaval, aortocaval, celiac, paraaortic, mesenteric, and peripancreatic regions (Figs. 3, 4). Distant lymphatic dissemination can occur in the mediastinum, cardiophrenic nodes and pelvis (Fig. 4). Regional and distant lymphadenopathy, especially with a lack of extranodal recurrence sites, needs to be identified as being different from PTLD, infectious conditions, or metastasis from a malignancy other than HCC. Vascularity, heterogeneity, and central necrosis should raise concerns for malignancy. The hyperenhancement on arterial phase contrast-enhanced CT or MR imaging seen in the nodes indicates that the origin is certainly from HCC.

### Peritoneal and/or omental recurrence

**Fig. 5:**
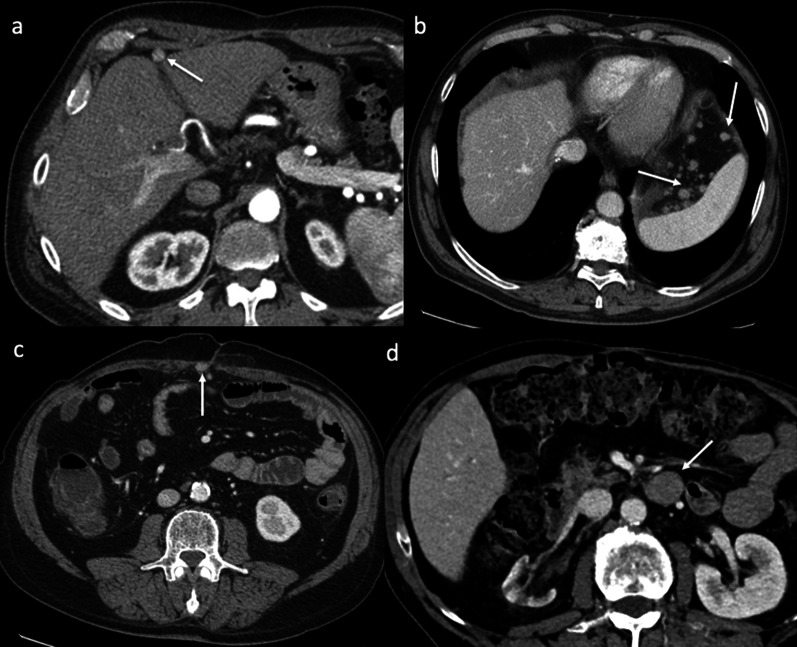
Peritoneal recurrence. Axial contrast-enhanced CT images (**a**–**d**) show different patients with peritoneal HCC recurrence (arrows), characterized by single or multiple discrete masses located in the peritoneal cavity or omentum. These intraperitoneal masses are often hypervascular on the arterial phase, such as the primary hepatic mass

**Fig. 6:**
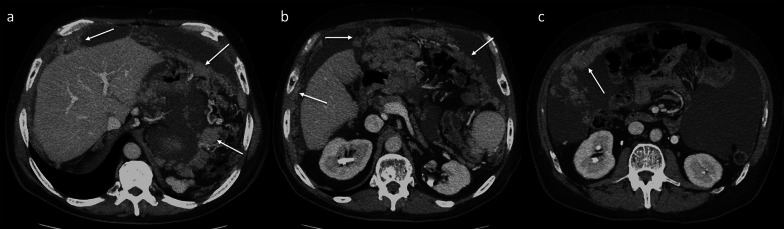
Omental recurrence. Axial contrast-enhanced CT images on the portal venous phase (**a**–**c**) show multiple confluent masses located in the peritoneal cavity and omentum (arrows). These lesions coalesce to form a diffuse soft tissue density mass, the so-called omental cake

The peritoneum and omentum are typical sites of HCC recurrence, via neoplastic spread through tumor cells in ascites or variceal collaterals (hematogenously) or by direct invasion (in particular from an exophytic lesion). The spread can also arise during liver transplantation from accidental rupture of the lesion with the spilling of tumor cells or from lymphatic leakage during liver hilum dissection. At imaging, the intraperitoneal dissemination of HCC appears as single or multiple enhancing masses located in the peritoneal cavity and/or omentum (Fig. 5). These intraperitoneal lesions are often hypervascular, such as the primary hepatic mass, and are most commonly supplied by the omental branches. Therefore, they can show hyperenhancement on arterial phase contrast-enhanced CT and MR imaging. Early omental disease shows itself in imaging as a permeated or smudged appearance of the omental fat and then progresses to enhanced soft tissue nodule shaping as the disease becomes more severe. Ultimately, these nodules come together to form a diffuse soft tissue mass that can displace the bowels, the so-called omental cake (Fig. 6). Malignant ascites may also be present. Peritoneal and omental HCC recurrence need to be differentiated from metastatic malignancies such as gastric, ovarian, or colon cancer.

### Adrenal gland recurrence

**Fig. 7:**
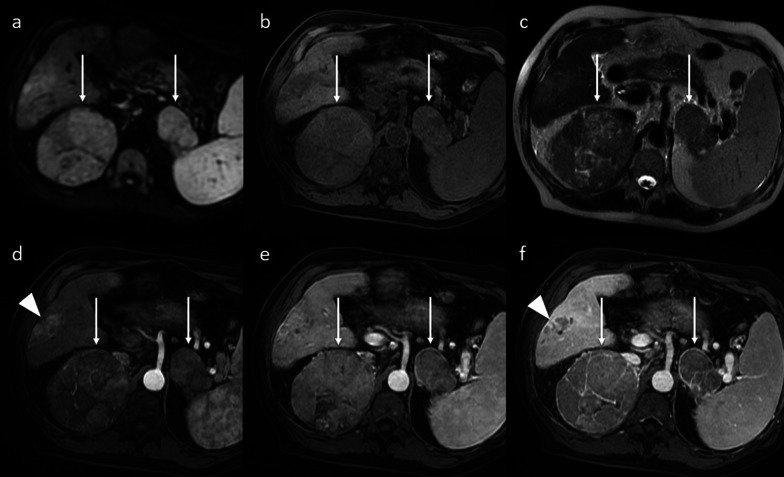
Adrenal gland recurrence. Axial MR imaging shows two masses in both adrenal glands (arrows). These lesions exhibit hyperintensity in DWI (**a**), hypointensity in a T1-weighted sequence (**b**), and inhomogeneous hyperintensity in a T2-weighted sequence (**c**). After contrast-medium administration, they appear slightly and nonhomogeneously hypervascular on the arterial phase (**d**) with washout and incomplete pseudocapsule in the portal venous (**e**) and 3-min late phases (**f**). Notice the coexistence of recurrent HCC in the liver with hyperenhancement on the arterial phase and washout in the late phase (arrowheads)

After lung and bone metastases, the adrenal gland is one of the most common methods of hematogenous dissemination. Adrenal metastases can be the first demonstration of extrahepatic recurrent HCC after LT. They can be found in only one or in both adrenal glands. However, the presence of an adrenal mass might not necessarily represent tumoral recurrence in patients who have undergone LT for HCC. Indeed, even with known primary HCC, adrenal enlargement can be an adenoma. For this reason, the comparison with previous imaging studies assumes particular importance. Strong enhancement during the arterial phase can be used to distinguish HCC from adrenal adenoma. Indeed, the adrenal metastasis from HCC commonly appears hypervascular on arterial phase contrast-enhanced CT and MR imaging (Fig. 7). However, hypoattenuation/hypointensity can be found in adrenal metastases from primary tumors other than HCC [16].

### Lung recurrence

**Fig. 8:**
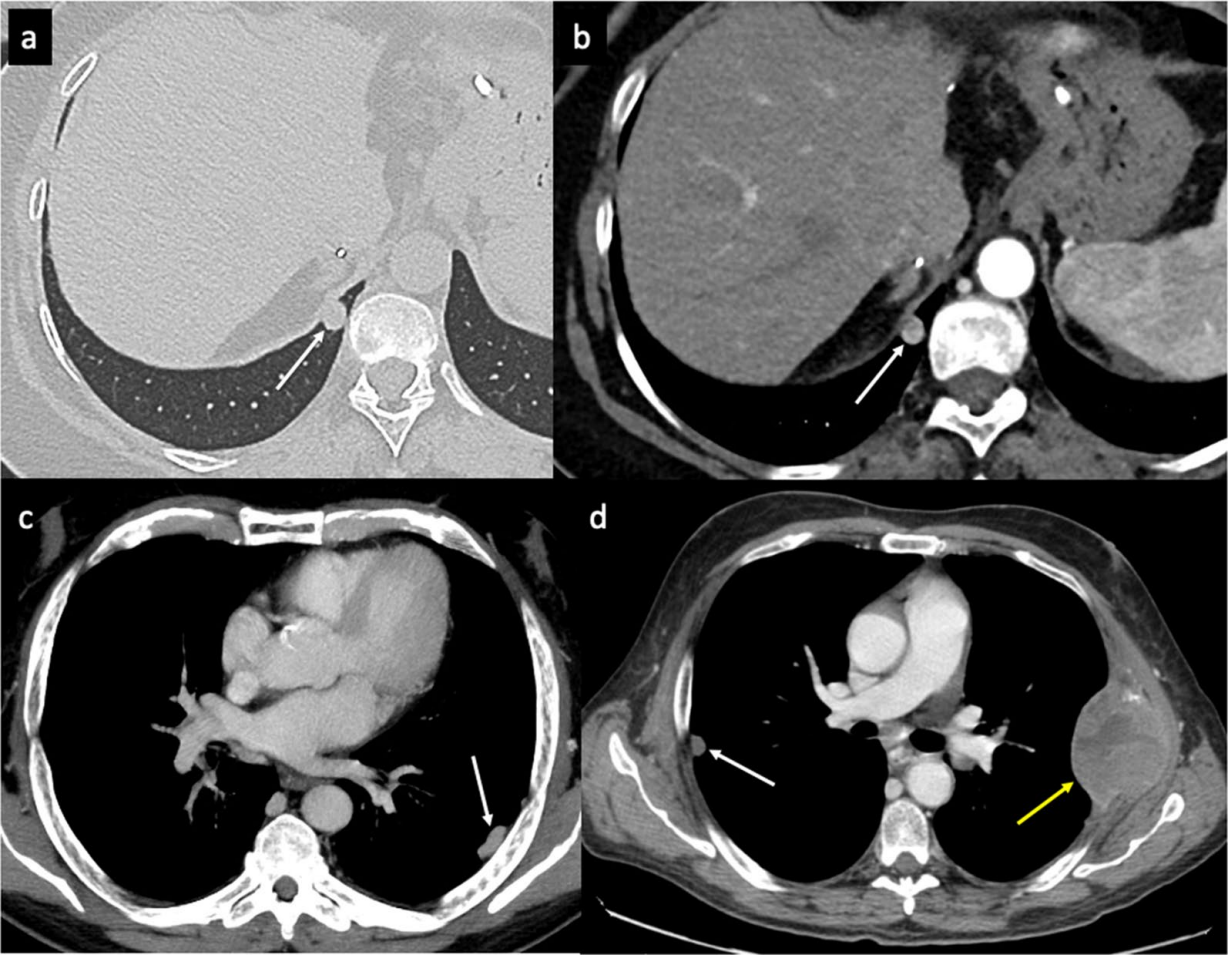
Lung and pleural recurrence. Axial unenhanced (**a**) and contrast-enhanced CT images on the arterial phase (**b**) exhibit a small lung recurrence in the right lower lobe (arrows). Notice the presence of hyperenhancement on the arterial phase due to the hypervascularity of the primary tumor. Axial contrast-enhanced CT images (**c**, **d**) show different pleural recurrences (white arrows) with the appearance of plaques or nodules. Notice the coexistence of rib metastasis (yellow arrow) with bone erosion

The lungs represent the most common site of recurrent HCC. The likely mechanism of tumor cells spreading to the lung is hematogenous dissemination through the pulmonary capillary network [15, 16]. Embolization of cancer cells via the hepatic veins may happen before or during LT, resulting in micrometastases being trapped within the capillary network of the lungs and nodule growth being increased by postoperative immunosuppression therapy. As is well known for hematogenous metastases from extrathoracic malignancies, the lower lung segments are seeded with tumor emboli more frequently than the upper areas. Lung metastases from HCC may be unique or multiple, involving one or both lungs. Lung recurrence is generally seen in the form of noncalcified enhancing soft tissue lesions within the lung parenchyma (Fig. 8), showing the same CT appearance as lung metastases from other primary tumors. Furthermore, pleural metastasis from HCC may be associated with lung recurrence (Fig. 8). During the acquisition of the arterial phase on chest CT, hyperenhancement of these lesions can be shown due to the hypervascularity of the primary tumor. Regarding differential diagnosis, lung nodules may represent metastases from a different malignancy, mycotic or opportunistic infections, a new primary lung malignancy, and/or PTLD [15]. In addition to hematogenous spread, recurrence may be located in the diaphragm since HCC can directly infiltrate it before LT.

### Bone recurrence

**Fig. 9:**
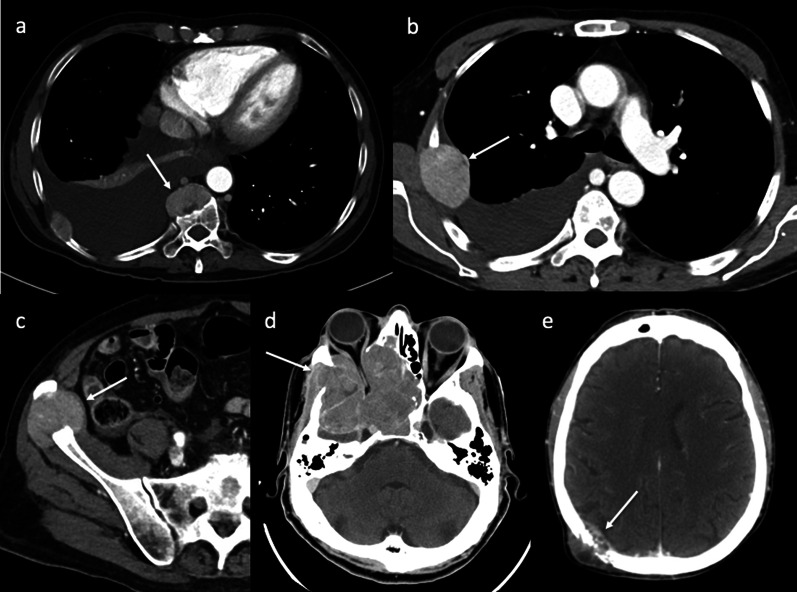
Bone recurrence. Axial contrast-enhanced CT images on the arterial phase (**a**–**e**) show bone recurrence of HCC (arrows) in different patients. Bone metastases from HCC are typically lytic (bone destruction), expansive, and characterized by soft tissue mass with typical hyperenhancement on the arterial phase. The sites of recurrence are vertebrae (**a**), ribs (**b**), right iliac bone (**c**), and skull (**d**, **e**). In patients with recurrent HCC in the right side of the anterior and middle cranial fossae (**d**), the lesion causes bone destruction with invasion of the orbit and ethmoid

One of the most frequent extrahepatic recurrences of HCC after LT is bone recurrence. Bone metastases may be isolated or multiple. The most common locations are in the axial skeleton, in particular in vertebrae, ribs, and the skull. Patients with bone recurrence can present with skeletal pain or pathologic fractures. Thoracic spine metastases may cause the collapse of vertebral bodies with extension into the spinal canal, which results in severe cord compression. Bone metastases from HCC are typically lytic (bone destruction), expansive, and characterized by soft tissue mass with typical hyperenhancement on arterial phase contrast-enhanced CT and MR imaging, as in the primitive tumor (Fig. 9) [16]. These imaging findings help to differentiate recurrent HCC from other primary tumor metastases.

### Brain recurrence

**Fig. 10:**
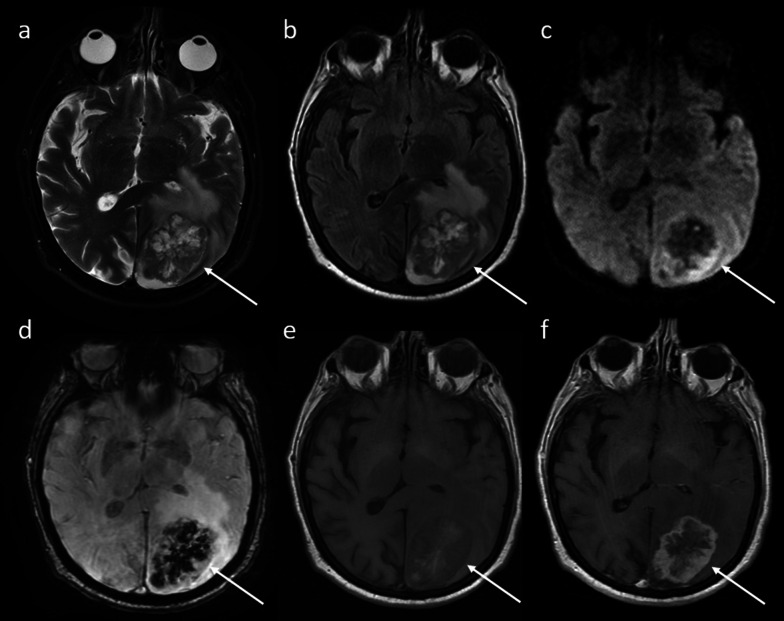
Brain recurrence. Axial MR imaging exhibits a left cerebral lesion (arrows) associated with perilesional edema. The lesion shows inhomogeneous hyperintensity in T2 (**a**), FLAIR (**b**), and DWI (**c**), and hemorrhagic changes in susceptibility-weighted sequence (**d**) and T1 (**e**). This brain recurrence of HCC appears as a ring-enhancing lesion in contrast-enhanced MR imaging (**f**)

The brain is an uncommon but possible site of HCC recurrence through hematogenous dissemination. Brain lesions are usually found after an initial manifestation of extrahepatic HCC at other more common sites. They may be solitary or multiple. Intracranial metastases usually look like parenchymal lesions, rarely presenting with leptomeningeal seeding. The hematogenous origin of these metastases is suggested by a gray–white matter junction and watershed zonal predominance. Brain recurrence of HCC seems to be a ring- or total enhancing lesion in contrast-enhanced CT and MR imaging (Fig. 10). Perilesional edema may be associated similarly with metastasis from other primary tumors. In addition, hemorrhagic changes may be shown reflecting the tumor hypervascularity of these lesions (Fig. 10). With regard to the differential diagnosis, brain HCC recurrence needs to be differentiated from cerebral abscess, PTLD, primary central nervous system tumors (lymphoma, glioblastoma), and metastases from a different malignancy.

### Soft tissue recurrence

**Fig. 11:**
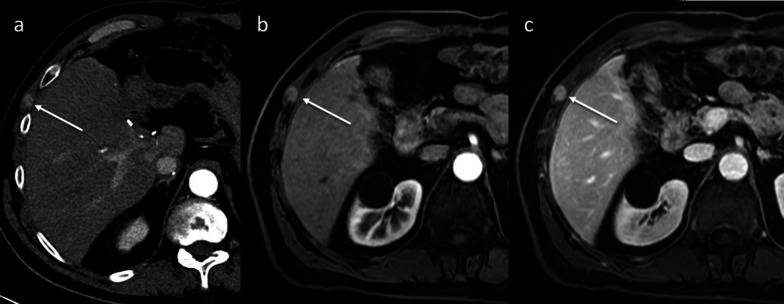
Soft tissue recurrence. Axial contrast-enhanced CT image on the arterial phase (**a**) exhibits a small muscular recurrence of HCC on the right abdominal wall, showing the typical hypervascular pattern (arrow). This lesion (arrows) is also confirmed on the arterial phase (**b**) and portal venous phase (**c**) contrast-enhanced MR images

**Fig. 12:**
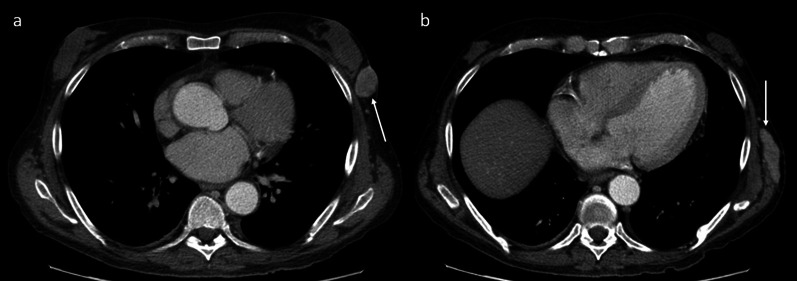
Soft tissue recurrence. Axial contrast-enhanced CT images on the arterial phase (**a**, **b**) exhibit recurrences of HCC involving the soft tissues of the left thoracic wall (arrows). These lesions show the typical pattern of the primary HCC, characterized by hyperenhancement on the arterial phase

Chest and abdominal wall involvement is an unusual site for HCC recurrence after LT. It can be the first presentation of metastatic disease with a solitary lesion, or it can be associated with metastases in other organs. HCC may spread to the chest and abdominal wall hematogenously or through lymphatic spread or direct invasion from an exophytic cancer. HCC recurrence can cover the soft tissues or muscles with a nodular appearance and often shows the typical pattern of the primary HCC on contrast-enhanced CT and MR imaging with hyperenhancement on the arterial phase (Figs. 11, 12).

## Recurrent HCC therapy

The detection of extrahepatic metastasis from HCC is crucial for treatment planning. The same treatment options currently available for HCC are also feasible for recurrent HCC after LT. The dissemination of the disease, either hepatic or extrahepatic, is of paramount importance in the choice of suitable treatment. The staging of these patients is based on CT of the chest and CT or MRI of the abdomen and pelvis and bone scintigraphy. However, since bone metastases from HCC are mostly osteolytic, the role of bone scintigraphy is debatable due to the lack of sensitivity for osteolysis. In up to 30% of patients, surgical resection should be the first option of treatment regardless of location, if technically feasible [10]. When surgery is not feasible, ablation has an important role in the treatment of small HCC recurrences in the liver because the results are similar to surgical resection [10]. Other locoregional treatment options are transarterial chemoembolization (TACE) and transarterial radioembolization (TARE), while systemic therapy with sorafenib should be considered for advanced cases if curative treatments are unfeasible. In this scenario, survival after recurrence decreases significantly [10]. Ultimately, the best supportive care should be offered to patients not eligible for any treatment.

## Conclusion

LT is the best curative option for patients with HCC and underlying liver cirrhosis. Nevertheless, organ shortages impose restrictive criteria and careful selection of patients. The evolution of clinical and molecular predictors would enable the inclusion of patients exceeding the conventional criteria who would have good outcomes. Nevertheless, when the Milan selection criteria are adopted, the risk of HCC recurrence is reported to be 8–20%. Therefore, any extended selection criteria with an increase in size and nodule number could lead to an increased risk of HCC recurrence. HCC recurrence can be located in the liver or be extrahepatic or both. Differential diagnosis between the recurrence of HCC and other malignant or benign lesions can and should be made using arterial phase enhancement, when present. Prompt detection of recurrent HCC after LT may facilitate resection of recurrent cancers or may enable timely decisions regarding alternative procedures or palliative treatment to be made. Therefore, radiologists should be aware that HCC can recur after LT with multiple organ involvement, and they should be familiar with the spectrum of disease.

## Data Availability

Data and material are available.

## Abbreviations

AFP: Alpha-fetoprotein
CNI: Calcineurin inhibitor
CT: Computed tomography
HCC: Hepatocellular carcinoma
LI-RADS: Liver Imaging Reporting and Data System
LT: Liver transplantation
MRI: Magnetic resonance imaging
mTOR inhibitors: Mammalian (mechanistic) target of rapamycin inhibitors
NASH: Non-alcoholic steatohepatitis
PTLD: Post-transplant lymphoproliferative disorder
TACE: Transarterial chemoembolization
TARE: Transarterial radioembolization
